# Anti-oxidative enzymes, cardiovascular disease risk factors, and adipokines in Nigerian women on oral and implant contraceptives

**DOI:** 10.4314/ahs.v25i1.22

**Published:** 2025-03

**Authors:** Bassey Iya, Uwem Akpan, Glory Nja, Ekaette Enang, Oglewu Adoga, Aniekan Etokidem

**Affiliations:** 1 University of Calabar, Department of Clinical Chemistry and Immunology, Faculty of Medical Laboratory Sciences University of Calabar; 2 University of Calabar, Department of Public Health, Faculty of Allied Medical Sciences, University of Calabar, Nigeria; 3 University of Calabar, Department of Statistics, Faculty of Physical Sciences. University of Calabar, Nigeria; 4 University of Calabar, Department of Community Medicine, University of Calabar Teaching Hospital, Calabar, Cross River State, Nigeria

**Keywords:** oral contraceptives, contraceptive implants, oxidative stress markers, lipid profile, adiponectin, leptin, black women

## Abstract

**Background:**

Increased use of oral contraceptives and implants has necessitated the need to examine biochemical changes associated with their use.

**Objectives:**

This study assessed insulin resistance, lipid profile, superoxide dismutase, glutathione peroxidase, adiponectin and leptin levels in women using oral and implant contraceptives in Calabar.

**Methods:**

Ninety women aged 18-45years were recruited for this case-control study. Thirty of them women were on oral contraceptives, 30 on implants and the remaining 30 were controls. Total cholesterol (TC), VLDL-cholesterol (VLDL-C), HDL-cholesterol (HDL-C), triglycerides, glucose, insulin, superoxide dismutase, glutathione peroxidase, adiponectin and leptin were analyzed by standard methods. Body mass index (BMI), Insulin resistance (HOMA-IR), Atherogenic index of plasma (AIP) and LDL-cholesterol (LDL-C) were calculated. Data was analyzed by Multivariate Analysis of Variance, Bonferrori Post Hoc test and Pearson's correlation.

Significance was set at <0.05.

**Results:**

The oral contraceptive users had significantly higher BMI (p=0.003), TC (p=0.004), HDL-C (p=0.000), Insulin (p=0.019), Leptin (p=0.000) and Adiponectin (p=0.049) than controls. Implant users had a significantly higher (p=0.000) BMI, TC, HDL-C LDL-C, Insulin, HOMA-IR, FPG (p=0.018) and Leptin (p=0.022) with significantly lower TG (p=0.003), VLDL-C (p=0.000) and AIP (p=0.003) compared to controls. There was a significant positive correlation between the duration of contraceptive use with LDL-C in implant users (r=0.366, p=0.046).

**Conclusions:**

Oral contraceptive use may be associated with weight gain while implant use may lead to derangement in carbohydrate, lipid metabolism and weight gain. This suggests the need for appropriate client profiling before recommending a contraceptive method, especially for those with pre-existing conditions.

## Introduction

Despite the high unmet need for family planning (19% for married and 48% for unmarried women) and the low uptake of modern contraceptive use in Nigeria (17% for married and 28% for unmarried women), it cannot be denied that the increased availability and awareness of different types of contraceptives has bestowed upon the Nigerian woman the ability to make definite choices^1^,^2^. Contraception also has offered them numerous amazing life-saving benefits including better postpartum health outcomes^3^. The current increase in the availability of contraceptive options has made possible the choice of using either Short-acting reversible contraceptives (SARCs) or Long-acting reversible contraceptives (LARCs).

SARC is defined as “contraceptive methods that require administration once or more per cycle or month and include barrier methods such as condoms, oral contraceptive pills, transdermal patches, and vaginal rings”^4^. LARC is defined in National Institute for Health and Care Excellence (UK) 2019 guideline as “contraceptive methods that require administration less than once per cycle or month”. LARCs include progestogen-only intrauterine systems; progestogen-only subdermal implants, copper intrauterine devices; as well as progestogen-only injectable contraceptives^5^. Compared to SARCs, LARCs are very convenient, highly effective, long-lasting, cost effective and has much better compliance rates. They also have the advantage of being able to reduce the gap between “perfect use” and “typical use” failure rates since there are no user-related errors^2^.

Despite these advantages, it has been documented that women prefer to use SARCs over LARCs for contraception, especially if they have not had children in order to avoid the stigma of being perceived as being infertile^2^,^6^. Most family health programmes globally are trying to encourage the use of LARCs as a main way of contraception, even in adolescent females. Though the logic behind this development is well thought out, it is pertinent to look at biochemical changes that are associated with long-term use of contraceptives (as opposed to short-term use) that may encourage the development of complications associated with contraceptive use and perhaps suggest ways to mitigate them. The SARC of interest in this study is Combined oral contraceptive (COC) while the LARC used is Implanon. Studies have shown that contraceptives like any other form of drug therapy lead to the release of free radicals in the body and these free radicals over time if not made stable by antioxidants lead to oxidative stress^7^.

Dyslipidaemia, fat accumulation and obesity (which are linked to an increased risk for cardiovascular disorders) have been associated with enhanced oxidative stress. Some studies have reported positive correlation of biomarkers of oxidative stress and negative correlation of biomarkers of an effective antioxidant system with body mass index^8^. It has also been reported that free radicals can cause increased adipogenesis leading to alterations in how adipocytokines such as leptin and adiponectin are produced^9^. Thus, it appears that there is an intrinsic relationship between obesity, dyslipidemia and oxidative stress. It would be important to explore the association between oxidative stress markers, adipokines and cardiovascular disease risk factors in women who take contraceptives. Studies in this subject area, especially in the black populace are scarce. Most of the studies available were carried out in Caucasian populations. There are also very few studies, if any, that compare anti-oxidative enzymes, cardiovascular disease risk factors and adipokines and the relationship between these parameters in women taking Oral and Implant contraceptives even in Caucasian populations. This study therefore assessed the effects of Oral and Implant hormonal contraceptives on fasting plasma glucose, insulin, insulin resistance, lipid profile, superoxide dismutase, glutathione peroxidase, adiponectin and leptin levels in women taking them in Calabar metropolis, the aim of which is to determine the relationships between these parameters. Null Hypothesislevels of women on who use them.

## Methods

### Description of the study setting

This study was conducted in Calabar, the state capital of Cross River State, Nigeria. Cross River State is one of the six states in the Ssouth-Ssouth region of Nigeria, located within the rain forest belt of the country. The State has an area of 20,156 square kilometres with an approximated population of 3,737,517 inhabitants as of 2016 national census^10^ Cross River State is presently a tourist attraction because it plays host to the whole world in its biggest street party “The Carnival Calabar” held annually in Calabar Municipality the State capital. The State comprises 18 Local 93 Government Areas (LGAs) divided into three senatorial districts (North having 5 five LGAs; Central having 6 six LGAs, and South having 7 seven LGAs). The State shares boundary with Benue State to the North, Akwa Ibom State to the South, Ebonyi State to the North West, Abia and Anambra States to the West, and the Republic of Cameroun to the East. There are three major ethnic groups in the State: Efik, Bekwarra, and Ejagham. Calabar Municipality the state capital lies between Latitude 04° 15^1^ and 5°N, and 50 E. It has an area of 406 square kilometres with an estimated 371,022 inhabitants and comprises ten political wards. Major occupations of the indigenes are fishing, farming and trading. There are two Tertiary health facilities (University of Calabar Teaching Hospital and Navy Hospital, Calabar); one Secondary health facility (General Hospital Calabar); and a host of privately own health facilities in the State capital.

### Study design and subject selection

#### Study design and sampling technique

A case-control design was used for the study. A total of 90 apparently healthy premenopausal females aged between 18 and 45 years (60 contraceptive users and 30 non-contraceptive users) were randomly recruited for the study. The contraceptive users were recruited from the University of Calabar Teaching Hospital and General hospital family clinic health facilities Family Health Clinics, while the non-contraceptive users were recruited from schools, church meetings, business places and offices within the study setting. The female contraceptive users were further divided into two groups: 30 women using combined oral contraceptive pills and contraceptive implants (Implanon NXT). A semi-structured standard questionnaire was used to obtain sociodemographic information.

## Ethical considerations

Ethical clearance was obtained from the University of Calabar Teaching Hospital Ethical research committee (UCTH/HREC/33/Vol.III/012). A form that asked questions such as requesting for brief description of our work, source of funding, benefit/harm to the patients, if the patients were to pay for any of the tests we were offering; how we were going to disseminate the information from the study to the participants was filled by the principal investigator. The proposal was attached to the form which was then assessed by the Ethical research committee and approval given after necessary corrections were made. The nature and purpose of this research were explained to the participants and each of them gave a written informed consent before being enrolled in the study.

### Sample size calculation

Sample size and power calculations for the Unmatched Case-Control study were done using the StatCalc function of Epi Info software by US Centre for Disease Control and Prevention (CDC). A two-sided confidence level of 99.9%, desired Power of 99%, the ratio of controls to cases as 1:2 and odds ratio of 209 were utilized and the percentage outcome in the unexposed group was 77% using data from a study by Cauci et al.,^11^. The Kelsey formula gave a sample size of 27 for controls and 54 for cases. Taking into cognizance an attrition rate of 10%, the sample size increased to 30 for controls and 59 for cases (i.e. a total of 89 participants). However, a total of 90 women were recruited comprising 30 non-contraceptive users and 60 women on contraceptives.

### Inclusion and Exclusion criteria

All subjects consisted of pre-menopausal women between the ages of 18 to 45 years who were consistently using oral contraceptives or contraceptive implants for at least six months.

All post-menopausal women, pregnant women, women who were smokers or consumed alcohol and antioxidant supplements, as well as women with any systemic disease, were excluded from the study.

### Instruments for data collection

#### Quantitative data collection

A semi-structured standard questionnaire containing question on age, education, marital status, parity, occupation, signs/symptoms, duration of Contraceptive use was used to obtain sociodemographic information from eligible study participants.

#### Sample collection

Five millilitres (5ml) of venous blood was collected aseptically from each subject by veniepuncture: 2mls was dispensed into a fluoride oxalate bottle and 3mls into a dry plain container at between 8.00am and 10.00am. The samples in the plain bottle were allowed to clot and then spun in a centrifuge at 3,000 revolutions per minute (rpm) for 5 minutes to obtain serum, while samples in the fluoride oxalate was spun and plasma obtained. The serum and plasma obtained were transferred into correctly labelled bottles and stored frozen at -200C until the time of analysis.

### Assay methods

#### Anthropometric and blood pressure measurements

The height and weight of the women were measured as described by Akpan et al.^12^. The body mass index was calculated as a ratio of weight (in kg) per height (in meters) squared. The Omron M2 Basic Blood Pressure Monitor was used to measure the blood pressure of the participants 15 min after they reached the clinic. The ensuing measurements were done after 5 min. The mean systolic and diastolic blood pressures were calculated as recommended by WHO^13^.

#### Estimation of lipid profile and plasma glucose

Lipid profile and glucose were analyzed from serum and plasma respectively by Selectra Pro XL Automatic Biochemistry analyzer (ELITECH, USA) at the Chemical Pathology Laboratory, glutathione peroxidase Insulin, leptin, adiponectin, superoxide dismutase and glutathione peroxidase were determined using ELISA kits from Sunlong Biotech, Hangzhou China according to the manufacturer's instructions.

#### Determination of Insulin Resistance

Insulin resistance was determined using the Homeostatic model assessment of insulin resistance (HOMA-IR) which is calculated as follows; HOMA–IR = (concentration of insulin x concentration of glucose)/22.5 for glucose concentration in mmol/L and insulin concentration in µIU/mL

### Statistical Analysis

The R version 4.0.0 (R Foundation for Statistical Computing Platform, Vienna, Austria) and PAWstatistic 25, (SPSS Inc, Chicago IL, USA) were used to analyze the data. Categorical variables are expressed as frequencies and continuous variable are presented as mean ± standard deviation. Chi-squared test of independence was used to test significant relationship between categorical variables. A one-way multivariate analysis of variance (MANOVA) was conducted to investigate if there was a significant difference in contraceptive effects on women based on different groups (control, oral and implant). An initial exploratory analysis of the dataset was carried out. The results showed that most of the variables were not normally distributed. A log transformation was done for those variables and the variables passed the normality test and all the other basics assumptions. The histograms, the Shapiro Wilk's test result, box plots, and other plots used in test for normality of the variable are attached in the S1 Appendix. After the rejection of the null hypothesis. A follow-up univariate analysis of variance (ANOVA) was carried out on each of the dependent variable, to ascertain which of them led to the rejection of the MANOVA null hypothesis. Bonferrori test was used for post-hoc analysis. Pearson's correlation was used to find the relationship among variables. The level of significance was set at 0.05 with 95% confidence interval. Aa p-value of less than 0.05 (p<0.05) was considered statistically significant.

## Results

Participants' characteristic, contraceptive use and commonly reported side effects

The demographic characteristics of the participants in the study are shown in Table 1. Majority of the women on oral contraceptives 18 (60.0%)(60.0%) and controls 26 (86.7%)(86.7%) had tertiary education. Oral contraceptives were more frequently used by single women and implants by married women. From the implant group, a majority 21 (70.0%) (70.0%) had only primary education.

**TABLE 1:** Demographic characteristics of the contraceptive and non-contraceptive users

Respondents' variables		Oral N = 30	Implants N = 30	Controls N = 30
Characteristics		n (%)	n (%)	n (%)
**Age (years)**	Mean±SD	25.30±6.01	25.80±3.89	24.70±6.75
**Marital status**	Single	23 (76.7)	4 (13.3)	22 (73.3)
	Married	7 (23.3)	26 (86.7)	8 (26.7)
**Education**	Primary	11 (36.7)	21 (70.0)	1 (3.3)
	Secondary	1 (3.3)	3 (10.0)	3(10.0)
	Tertiary	18 (60.0)	6 (20.0)	26(86.7)
**Occupation**	Manual and semiskilled laborers	3 (10.0)	0 (0.0)	0(0.0)
	Civil or public servants	5 (16.7)	14 (46.7)	3(10)
	Business	13 (43.3)	12 (40.0)	5(16.7)
	Student	7 (23.3)	0 (0.0)	22 (73.3)
	Unemployed	2 (6.7)	4 (13.3)	0 (0.0)
**Signs/symptoms**	Weight gain	13 (43.3)	0(0.0)	0(0.0)
	Irregular menses	3 (10.0)	0(0.0)	0(0.0)
	Spotting	5 (16.7)	7(23.3)	0(0.0)
	Headache	1 (3.3)	6 (20.0)	0(0.0)
	Increased menstrual bleeding	0 (0.0)	6 (20.0)	0(0.0)
	UTI	0 (0.0)	2(6.7)	0(0.0)
	None	8 (26.7)	9(30.0)	0(0.0)
**Contraceptive use**	Yes	30 (100.0)	30 (100.0)	0 (0.0)
	No	0 (0.0)	0(0.0)	30 (100.0)
**Duration of use (years)**	Mean ± SD	1.61±1.37	3.47±1.43	0(0.0)

Business women constituted the highest percentage of oral contraceptive users^13^ (43.3%)(43.3%) while civil servants were more 14(46.7%)(46.7%) among the implant users. For the control group, students were more accounting for 22 (73.3%)73.3% of the population. For women on oral contraceptives, the three most reported side effects were weight gain13(43.3%)(43.3%), irregular menses 5 (16.7%)(16.7%) and spotting 3 (10.0%)(10%); while for the implant users, it was spotting 7(23.3%)(23.0%), headache 6 (20.0%)(20.0) and increased menstrual bleeding 6 (20.0%)(20.0%). The mean duration of contraceptive use for oral contraceptive and implant users was 1.61±1.37years and 3.47±1.43 years respectively. Participants' contraceptive use and effect on body mass index, blood pressure and measured blood parameters.

Results of the MANOVA presented in Table 2 showed a statistically significant difference in the mean effects of contraceptive use across the three groups (control, oral and implant) based on the combined dependent variables (Wilk's lambda = 0.105, F(36,140) = 8.138, p < .05, partial *η*2 = 0.677, observed power = 1.00). The evidence was thus sufficient to reject the null hypothesis and conclude that the mean effect of contraceptive use on the dependent variables among women is significantly different across the three groups. The effect size was large. The observed power was 1.00, indicating that there was a 100% chance or accuracy that the results could have come out significant.

**Table 2:** MANOVA output for contraceptive Effects across the groups

Statistic	Value	*F*	Hyp. *Df*	Error *df*	*p*-value	Partial h^2^	Observed power
**Pillai's Trace**	1.324	7.725	36.000	142.000	0.000		1.00
**Wils' lambda**	.105	8.138	36.000	140.000	0.000		1.00
**Hotelling's trace**	4.466	8.560	36.000	138.000	0.000		1.00
**Roy's largest root**	3.175	12.523	18.000	71.000	0.000		1.00

A follow-up univariate analysis of variance (ANOVA) was carried out on each of the dependent variables (body mass index, blood pressure, lipid profile, atherogenic index of plasma, fasting plasma glucose, insulin, HOMA-IR, leptin, adiponectin, superoxide dismutase and glutathione peroxidase) to ascertain which of them led to the rejection of the MANOVA null hypothesis. The results are shown in Table 3 and 4. Table 3 shows the body mass index, blood pressure, lipid profile, atherogenic index of plasma, fasting plasma glucose, insulin, HOMA-IR, leptin, adiponectin, superoxide dismutase and glutathione peroxidase among contraceptive users and controls while the Table 4 shows the values of the log-transformed values of the above mentioned variables. There were significant variations (p=0.0001) in the mean effect of contraceptive use on the levels of BMI, Diastolic BP, triglycerides, Total cholesterol, HDL Cholesterol, LDL-Cholesterol, VLDL-Cholesterol, atherogenic index of plasma, Fasting plasma glucose, Insulin, HOMA-IR, Leptin and Adiponectin among the groups. There were no significant variations (p>0.05) in the effect of contraceptives on the levels of superoxide dismutase and glutathione peroxidase among the groups.

Post hoc analysis in Table 5 showed that BMI was significantly higher in both oral contraceptive (p = 0.003) and implant groups (p = 0.0001) compared to the control group. There was no significant difference (p > 0.05) in BMI between the oral contraceptive and implant groups. Diastolic blood pressure was significantly lower in the oral contraceptive compared to the control (p = 0.008) and implant groups (p = 0.017).

**Fig 1:**
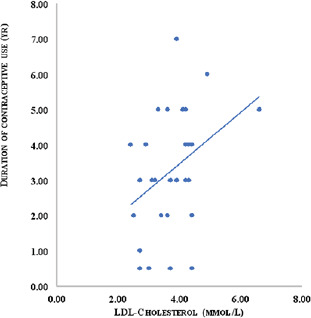
Correlation plot of duration of contraceptive use against LDL-cholesterol in women who use implant contraceptives

There was no significant difference (p > 0.05)in diastolic blood pressure between the control and implant groups. Triglyceride and VLDL-C were significantly lower in the implant group compared to the control (p = 0.003) and oral contraceptive (p = 0.0001) groups. There was no significant difference (p > 0.05) for both Triglyceride and VLDL-C between the control and oral contraceptive group. The implant group had the highest levels of Total Cholesterol (TC). TC was significantly higher in both oral contraceptive (p = 0.004) and implant groups (p = 0.0001) compared to the control group. The implant groups also had significantly higher TC compared to the oral contraceptive groups. Glucose was higher in women who used implants compared to the control (p = 0.018) as well as the those who used oral contraceptives (p = 0.001). However, no significant difference (p >0.05) was observed in glucose levels between the control and oral contraceptive users. Insulin was significantly higher in both oral contraceptive (p = 0.019) and implant users (p = 0.0001) compared to the control group. The women who used implants however had significantly higher HOMA-IR than the oral contraceptive (p = 0.011) and control (p = 0.0001) groups. There was no significant difference (p > 0.05) for insulin and HOMA-IR between the control and oral contraceptive group. The oral contraceptive users had significantly higher leptin levels compared to the controls (p=0.0001) and implant users (p = 0.041). The oral contraceptive users also had significantly higher leptin (p = 0.041) compared to the implant group. Similarly, the oral contraceptive users had significantly higher adiponectin levels compared to the control (p=0.003) and implant groups (p = 0.016). However, there was no significant difference (p > 0.05) in adiponectin between the control and implant groups. Fig 1 shows a significant positive (r=0.416, p= 0.001) correlation of duration of contraceptive usage with LDL-C.

**Table 3:** Mean body mass index, blood pressure, lipid profile, atherogenic index of plasma, fasting plasma glucose, insulin, HOMA-IR, leptin, adiponectin, superoxide dismutase and glutathione peroxidase among contraceptive users and controls

Parameter	Oral contraceptive users	Implant users	Controls	F-cal	P-value
	N=30	N=30	N=30		
**BMI (kg/m^2^)**	23.9±3.39	25.2±3.73	21.2±1.46	12.149	0.000**
**Systolic BP (mmHg)**	116.9±9.19	113.8±6.15	117.6±4.58	2.686	0.074
**Diastolic BP (mmHg)**	74.2±7.67	78.4±5.81	78.7±3.54	5.933	0.006**
**TG (mmol/l)**	1.38±0.51	0.87±0.29	1.15±0.32	14.413	0.000**
**TC (mmol/l)**	4.76±0.99	5.40±0.94	4.05±0.50	20.541	0.000**
HDL-C (mmol/l)*	1.39±0.22	1.29±0.24	0.99±0.29	20.330	0.000**
LDL-C (mmol/l)*	2.75±0.99	3.74±0.86	2.53±0.64	17.462	0.000**
**VLDL-C (mmol/l)**	0.63±0.23	0.40±0.13	0.52±0.15	14.480	0.000**
**AIP**	-0.04±0.22	-0.20±0.21	0.07±0.18	13.650	0.000**
**FPG (mmol/l)**	3.91±0.59	4.76±1.07	4.11±0.68	7.955	0.001**
**Insulin ((µIU/ml)**	27.67±45.61	62.02±79.32	10.13±7.39	12.872	0.001**
**HOMA-IR**	4.51±6.64	15.04±20.84	1.80±1.32	12.789	0.000**
**Leptin (pg/ml)**	1682.3±943.09	1146.1±678.13	938.6±911.31	13.844	0.000**
**Adiponectin(ng/ml)**	25.8±14.41	18.3±9.74	18.4±12.84	6.749	0.005**
**SOD(pg/ml)**	2597.4±2403.18	2002.8±1829.46	2146.2201.70	1.300	0.278
**GPx(pmol/ml)**	57.4±72.37	34.5±41.97	40.9±54.77	2.037	0.137

Values are expressed as Mean ± SD

Key: BMI-Body mass index, TG-Triglycerldes, TC-Total cholesterol, HDL-C High density lipoprotein, LDL-C Low density lipoprotein, VLDL-C Very low density lipoprotein, AIP- Atherogenic index of plasma

**Table 4:** Transformed dependent variable ANOVA Statistics among contraceptive users and controls

Parameter	Oral contraceptive users	Implant users	Controls	F-cal	P-value
	N=30	N=30	N=30		
**BMI**	1.37±0.61	1.39±0.06	1.32±0.05	12.149	0.000*
**Systolic BP**	2.07±0.03	2.06±0.23	2.07±0.02	2.686	0.074
**Diastolic BP**	1.87±0.05	1.89±0.32	1.89±0.02	5.933	0.006**
**TG**	0.11±0.16	-0.08±0.14	0.04±0.13	14.413	0.000**
**TC**	0.67±0.09	0.73±0.07	0.61±0.05	20.541	0.000**
HDL-C*	1.39±0.22	1.29±0.24	0.99±0.29	20.330	0.000**
LDL-C*	2.75±0.99	3.74±0.86	2.53±0.64	17.462	0.000**
**VLDL-C**	-0.23±0.16	-0.42±0.14	-0.29±0.13	14.480	0.000**
AIP*	-0.04±0.22	-0.20±0.21	0.07±0.18	13.650	0.000**
**FPG**	0.59±0.07	0.67±0.09	0.61±0.07	7.955	0.001**
**Insulin**	1.19±0.43	1.47±0.54	0.85±0.45	12.872	0.001**
**HOMA-IR**	0.45±0.36	0.79±0.59	0.22±0.26	12.789	0.000**
**Leptin**	3.18±0.20	3.01±0.19	2.84±0.33	13.844	0.000**
**Adiponectin**	1.37±0.19	1.22±0.16	1.19±0.22	6.749	0.005**
**SOD**	3.30±0.29	3.19±0.28	3.20±0.29	1.300	0.278
**GPX**	1.57±0.35	1.43±0.25	1.44±0.33	2.037	0.137

* Not transformed because they passed the Shapiro-Wilk's normality test

** significant at p<0.05

Key: BMI-Body mass index, TG-Triglycerides, TC-Total cholesterol, HDL-C High density lipoprotein, LDL-C Low density lipoprotein, VLDL-C Very low density lipoprotein, AIP- Atherogenic index of plasma

**Table 5:** Post hoc analysis of body mass index, blood pressure, lipid profile, AIP, fasting plasma glucose, HOMA-IR, insulin, leptin, adiponectin among contraceptive users and controls

Parameter	Oral contraceptive users N=30	Controls N=30	Mean Diff	p-value
**BMI (kg/m^2^)**	1.37±0.61	1.32±0.05	0.052	0.003
**TC (mmol/l)**	0.67±0.09	0.61±0.05	0.064	0.004
**HDL-C (mmol/l)**	1.39±0.22	0.99±0.29	0.397	0.000
**Insulin (µIU/ml)**	1.19±0.43	0.85±0.45	0.343	0.019
**Leptin (pg/ml)**	3.18±0.20	2.84±0.33	0.340	0.000
**Adiponectin(ng/ml)**	1.37±0.19	1.19±0.22	0.167	0.049

	**Implant users N=30**	**Controls N=30**		

**BMI (kg/m^2^)**	1.39±0.06	1.32±0.05	0.073	0.000
**TG (mmol/l)**	-0.08±0.14	0.04±0.13	-0.126	0.003
**TC (mmol/l)**	0.73±0.07	0.61±0.05	0.122	0.000
**HDL-C (mmol/l)**	1.29±0.24	0.99±0.29	0.298	0.000
**LDL-C (mmol/l)**	3.74±0.86	2.53±0.64	1.207	0.000
**VLDL-C (mmol/l)**	-0.42±0.14	-0.29±0.13	-0.125	0.003
**AIP**	-0.20±0.21	0.07±0.18	-0.271	0.000
**FPG (mmol/l)**	0.67±0.09	0.61±0.07	0.058	0.018
**Insulin (µIU/ml)**	1.47±0.54	0.85±0.45	0.622	0.000
**HOMA-IR**	0.79±0.59	0.22±0.26	0.562	0.000
**Leptin(pg/ml)**	3.01±0.19	2.84±0.33	0.177	0.022

	**Oral contraceptive users N=30**	**Implant users N=30**		

**Diastolic BP (mmHg)**	1.87±0.05	1.89±0.32	-0.025	0.018
**TG (mmol/l)**	0.11±0.16	-0.08±0.14	0.195	0.000
**TC (mmol/l)**	0.67±0.09	0.73±0.07	-0.058	0.004
**LDL-C (mmol/l)**	2.75±0.99	3.74±0.86	-0.987	0.000
**VLDL-C (mmol/l)**	-0.23±0.16	-0.42±0.14	0.195	0.000
**AIP**	-0.04±0.22	-0.20±0.21	0.159	0.009
**FPG (mmol/l)**	0.59±0.07	0.67±0.09	-0.080	0.001
**HOMA-IR**	0.45±0.36	0.79±0.59	0.333	0.011
**Leptin (pg/ml)**	3.18±0.20	3.01±0.19	0.162	0.041
**Adiponectin(ng/ml)**	1.37±0.19	1.22±0.16	0.139	0.016

Key: BMI-Body mass index, TG-Triglycerides, TC-Total cholesterol, HDL-C High density lipoprotein, LDL-C Low density lipoprotein, VLDL-C Very low density lipoprotein, AIP- Atherogenic index of plasma

## Discussion

In this study, the effects of hormonal contraceptives on Systolic blood pressure (SBP), diastolic blood pressure (DBP), fasting plasma glucose, insulin, lipid profile, adiponectin, leptin, superoxide dismutase and glutathione peroxidase were investigated in women using oral contraceptives and implants within Calabar metropolis. Though the SBP was similar across the three groups, the DBP was lower in the oral contraceptive users compared to both the implant and control groups. A probable mechanism by which DBP may be decreased in oral contraceptive users is attributable to its estrogen constituents. It is well well-known that both ER-independent and ER-dependent mechanisms can be utilized by estrogen to cause vasodilation^14^. This is facilitated partly through the generation of endothelium-derived Nitric oxide by inducing in endothelial cells; causing an increase in intracellular free calcium concentration^15^,^16^ which in turn increases endothelial-derived nitric oxide. This gives estradiol its protective capability against hypertension^17^.

In addition, vascular smooth muscle cells the production of adenosine in (via the cyclic AMP adenosine pathway) is also stimulated by estrogen^18^. Also, the induction of cyclooxygenase and prostacyclin synthase expression by estrogen also leads to an increase in the synthesis of prostacyclin; which is a vasodilator^19^.

Finally, the production of powerful vasoconstrictors such as endothelin-1, angiotensin II as well as catecholamines is reduced by estrogen^20^. All of these mechanisms lead to a reduction in blood pressure. Our findings are supported by observations by some authors who reported lower diastolic blood pressure in women taking estrogen/progestin contraceptives 21-23. Contradicting the findings of these study, some others have reported a steady increase in both SBP and DBP in oral contraceptive users^24^-^25^. However, a possible explanation for these differing effects of oral contraceptives on blood pressure may be due to the differences in age, and race/ethnicity of participants in the different research groups. Body mass index (BMI) was significantly higher in both oral users and implant users compared to the controls which is an indication of weight gain. The mechanisms for this are different for the two contraceptive groups. Weight gain in oral contraceptive use may be associated with its effect on the appetite regulation and expenditure of energy by reducing cerebrospinal fluid leptin levels or its glucocorticoid-like activity^13^. The progestins in the oral pills increase appetite by suppression of the satiating peptide – CCK's secretion and thus causing body fat accumulation, and consequently, weight gain in oral contraceptive users. Furthermore, estrogen facilitates the metabolism of lipids and accumulation of fat in the adipose tissue. Consequently, combined oral contraceptive users are at risk of being overweight or obese^26^. In contrast, a study by Bawah et al., 2018 reported no significant differences in BMI of oral contraceptive users and the controls^27^.

The BMI of implant users though higher than that of both the controls and oral contraceptive users was in the pre-obese category according to WHO classification^28^. A possible mechanism by which implants increase BMI is due to the actions of its progestin component.

In addition to the mechanism ascribed to progestin above, progestin also enlarges adipocyte cell size but cell number is not affected^29^. This is because it is able to stimulate lipoprotein lipase (LPL), an enzyme that promotes circulating plasma TG hydrolysis and free fatty acids uptake by fat cells, thereby enhancing depot triglyceride storage. Implants may also increase BMI through a progestin mediated increase in water retention apparently increasing weight without a change in body fat composition. Studies by Kapp et al., 2015 and Griffin et al., 2017 reported similar observations in women using implants^30^-^31^. However, contrary to these findings, a 2016 study reported that the weight gain, if any, attributable to hormonal contraceptive implant was minimal^32^. The oral contraceptive users had significantly higher TC and HDL-C while the implant users had significantly higher TC, LDL-C, HDL-C and lower VLDL-C and TG when compared to the controls. Though the TG and VLDC of the oral contraceptive group were not significantly higher than that of controls, it was higher than that of the implant group. Oral contraceptives utilize the genomic pathway to change the lipid levels, in which modifications by estrogen affect hepatic apolipoprotein regulation^25^. The estrogen component of COCs boosts the clearance of LDL cholesterol and while raising levels of HDL-C. Though estrogen has been shown to increase triglyceride levels modestly this may not increase the risk of atherogenesis due to the concomitant higher HDL-C and lower LDL-C^25^. The higher HDL-C levels among oral contraceptive users reported in this study are consistent with the findings of Guedes et al., (2018) who reported similar findings^33^.

They are contrary to those of Okere et al., 2012 and Asare et al., (2014) who demonstrated that oral contraceptives, did not induce any significant differences in the HDL-C levels in women who used them^25^,^34^. The higher levels of TC in oral contraceptive users may be due to the higher levels of HDL-C observed in that group. The higher mean TC observed in the oral contraceptive users is consistent with the findings of Guedes et al., (2018) which showed that oral contraceptive users have a significantly higher TC than non-users^33^. However, other cross sectional studies by Bawah et al., (2015) reported a non-significant difference in serum TC levels in women who use oral contraceptives compared to those who do not^5^. For the implants users, various side effects of progestins on the metabolism of lipids, such as reducing HDL-C, are said to be mediated through the activation of the androgen receptor.

Progesterone administration leads to decrement in HDL-C levels resulting in increased Scavenger Receptor B, class 1 (SR-B1) expression in hepatocytes as well as increased plasma hepatic lipase activity^13^. A rise in SR-B1 causes an enhancement of cholesterol transfer from HDL particles into the liver cells thereby causing decrease in HDL-C levels. However, the implant used by women in this study is Implanon. Implanon is a progestin only contraceptive that contains etonogestrel, which is a highly selective progestogen with great affinity for the progesterone receptor. Due to its anti-androgenic effects it produces the greatest improvements in the lipid profile. Etonogestrel upregulates sex hormone binding globulin, hence decreasing the bioavailability of circulating free androgen and decreasing SRB1 receptor expression leading to increased HDL-C concentrations.

The mechanism of how low-density lipoprotein (LDL) might be affected by progesterone administration is not clear. However it has been documented that progesterone's antagonizing effect on estrogen's ability to stimulate LDL receptor expression in the liver, may result in decreased LDL-C clearance and higher plasma LDL cholesterol levels^13^. The high levels of total cholesterol in implant users may be a consequence of the increased LDL-C levels. Progestin when used alone (not in combination with estradiol) lowers plasma triglycerides and VLDL-C levels due to enhanced fractional catabolism of VLDL as well as the inhibition of hepatic triglyceride synthesis by progestins by inhibiting, hepatic glycerol-3-phosphate acyltransferase^36^.

In this study, the correlation of duration of use with lipid profile among the implant users showed a significant positive correlation with LDL-C. indicating that LDL-C increases with longer usage of implants. High levels of LDL-C represent one of the independent risk factors for coronary heart diseases in humans. An excessive influx of LDL-C through the scavenger pathway could result in atheroma formation through the cholesterol deposition in walls of the arteries^37^.

Both contraceptive groups had higher Leptin than controls. Leptin is an adipocytokine mainly made by white adipose tissue. It facilitates energy homeostasis and body weight regulation^38^.

High levels are associated with an increase in adipocyte mass associated with weight gain. Prolonged high levels of leptin over time may result in a condition called “leptin resistance”, in which there is an attenuation of leptin signaling. This results in leptin losing its capacity to decrease body weight/adiposity, depress hunger and increase energy expenditure resulting in obesity and its complications^38^. However, a study by Fallah et al., (2011) documented no significant change in serum leptin levels of low-dose combined oral contraceptive users in Brazil^39^. Insulin is also a major regulator of leptin as such high levels of glucose (observed in both groups of contraceptive users) also result in high levels of leptin and may also contribute to the phenomenon of leptin resistance and consequent weight gain.

Interestingly, significantly higher levels of adiponectin were only seen in oral contraceptive users. The reason or mechanism for this is not well understood as an increase in body mass has been associated with a decrease in adiponectin. Adiponectin is a vital part of the adipovascular axis and confers protection on the blood vessels by its pleiotropic actions. It acts as a biologically relevant endogenous modulator of the response of endothelial cells to pro-inflammatory stimulation, reduces oxidative/nitrative stress, inhibits inflammatory response induction and induces endothelial nitric oxide production and vascular relaxation^40^. This may also be a contributory factor to the lower DPB and the lower number of CVD risk factors seen in the oral contraceptive users when compared to the implant group.

The oral contraceptive group had only higher BMI as compared to higher BMI, insulin resistance, TC and LDL-C seen in the implant group. Only insulin was higher in oral contraceptive while the implant contraceptive users had significantly higher FPG, insulin and HOMA-IR compared to controls. This observation may be attributed to differences in the composition of oral contraceptives and implants. COCs contain progestogens and oestrogen, while implants contain only progestogens. Both oestrogen and progestogens modify carbohydrate metabolism and the net effect on glucose use can be attributed to the balance of both components in any particular formulation^41^. Carbohydrate metabolism is improved by small amounts of estrogen and has been shown to improve hyperglycemia in non insulin dependent diabetes. This improvement may be probably due to the inhibition of insulin degradation or alterations of insulin receptor binding^41^. On the other hand, high doses of progesterone and moderate to low doses of synthetic progestogens may impairment of glucose utilization, insulin resistance and consequently, an increase in the risk of coronary artery disease.

The use of oral contraceptive pills has been linked to various side effects including an imbalance between antioxidants and oxidative stress. This may change liver enzyme levels that control lipid synthesis and/or turnover^42^.

Contraceptives has been shown to be pro-oxidant and enzymes such as SOD, GPx and CAT which detoxify ROS, represent a key defense mechanism in combating ROS^43^. The dismutation of superoxide radicals to hydrogen peroxide by SOD is the first line of defence against ROS. However, our study did not observe any change in both SOD and GPx activities. The reason for this is not clear, but could point to the fact that it may be the non enzymatic enzyme system that is used up by the ROS generated or the enzymatic system is able to regenerate itself as quickly as it is consumed. A study by Fallah et al.^44^, reported increased GPx concentrations in while SOD remained unchanged in women on Oral contraceptives. Also Kowalska et al.^45^, reported increased Catalase concentrations in while SOD remained unchanged in women on Oral contraceptive. This may be an indication that the SOD enzyme seems to be less sensitive to hormonal changes in contraceptive users.

This study has shown that apart from weight gain observed in both oral and implant users, the implant users show a tendency to dyslipidaemia which increases with duration of use as well as insulin resistance. It has been demonstrated that insulin resistance in women who use contraceptives causes a number of abnormalities in metabolic and regulatory processes, which in turn causes the accumulation of cholesterol. Dyslipidaemia is a risk factor for coronary heart disease. Insulin resistance may increase with an increase in weight gain which has been observed in both oral and implant users. All these factors can predispose contraceptive users to Type 2 diabetes mellitus and cardiovascular disease^25^. The results from this study also suggest that the use of oral contraceptives may be safer to adopt in family planning than the use of implants due to the fewer cardiovascular risk factors seen in this group compared to that seen in implant users. Also during the long and continuous periods of implant contraceptive use, it may be advisable to include glycemic and insulin resistance indices in their management plan to prevent complications associated with them.

Although our study is potentially of interest and a number of parameters have been considered, the limitations in our study include the fact that the sample size is small and therefore the small number of enrolled patients may not allow us exclude that the difference noticed in BMI in the different groups that could influence all other parameters independently from the kind of contraception used. However, since the age ranges of the women are statistically similar and their lifestyle, habits, and diet are similar being from the same locality, it suggests that the changes in Body mass index may be associated with the use of the different contraceptives.

## Conclusion

The findings of this study showed that the oral contraceptive users had significantly higher BMI, TC and HDL-C, leptin and adiponectin while the implant users had higher BMI, TC and HDL-C, leptin, LDL-C, glucose, insulin and HOMA-IR and lower TG and VLDL-C. This suggests that oral contraceptive use may be associated with weight gain while implant use may lead to derangement in carbohydrate, lipid metabolism and weight gain. This suggests the need for family planning service providers to undertake appropriate client profiling before recommending a particular method to women seeking their services in order to minimize adverse health risk, especially for those who may have pre-existing conditions like diabetes, obesity and hypercholesterolamia.
